# Seizure as an Atypical Presentation of New-Onset Multiple Sclerosis: A Case Report

**DOI:** 10.7759/cureus.80626

**Published:** 2025-03-15

**Authors:** Shadrack Ansong, Wayne Jang, Victor Okoro, Elizabeth Onyeaso, Chika Okafor

**Affiliations:** 1 Internal Medicine, Cape Fear Valley Medical Center, Fayetteville, USA; 2 Internal Medicine, Campbell University School of Osteopathic Medicine, Lillington, USA

**Keywords:** aed (anti-epileptic drugs), focal neurological seizures, mri brain and spine, multiple sclerosis exacerbation, seizures

## Abstract

Multiple sclerosis (MS) can present with a wide range of symptoms in affected individuals. We report the case of a 56-year-old African-American woman with a history of hypertension who was admitted to the hospital due to persistent generalized weakness and intermittent seizures. According to her son, these symptoms began after starting antihypertensive therapy. She had experienced multiple hospital admissions for intermittent weakness and received treatment for a UTI and ruptured acute appendicitis. During one admission, she was prescribed Keppra at a dose of 500 mg twice daily. However, her symptoms persisted with no significant improvement, leading to an increased Keppra dosage of 750 mg twice daily. Imaging studies, including CT scans from all admissions, showed chronic ischemic changes but no acute abnormalities. An EEG was normal, but an MRI of the head and cervical spine revealed lesions characteristic of MS, including white matter inflammation, demyelination, and scarring. Her symptoms resolved following treatment with a 500 mg pulse dose of steroids for an MS exacerbation. This case highlights that effective seizure management often improves with appropriate treatment of the underlying disease. Once MS was identified and treated, her seizures resolved, and she continued on 750 mg of Keppra.

## Introduction

The prevalence of multiple sclerosis (MS) is estimated to be 2.5 cases per 100,000 individuals annually, based on data from 75 countries [1,2]. Most individuals diagnosed with this condition present with various symptoms, including optic neuritis, focal neurological deficits such as neurogenic bladder and bowel incontinence, weakness in the lower and upper extremities that may result in a bedridden state, and sensory impairments [2,3]. Additionally, some patients experience internuclear ophthalmoplegia, a condition marked by impaired horizontal eye movements [1,3].

Although seizures can occur in various intracranial disorders, they are considered uncommon in MS and may delay the accurate diagnosis and treatment of acute exacerbations [1]. When seizures do occur in the context of MS, antiepileptic drugs (AEDs) may have reduced effectiveness, often requiring higher doses or combination therapy to achieve adequate symptom control [2,4]. However, effective management of MS can potentially prevent seizures with a single AED at a lower dosage.

We present a case of newly diagnosed MS that manifested as seizures in an atypical patient.

## Case presentation

We present a case involving a 56-year-old African American female who was admitted to the hospital due to altered mental status. The patient had a history of three previous admissions for acute encephalopathy, with no documented focal neurologic deficits on examination. During those hospitalizations, she received treatment for a UTI on one occasion, sepsis resulting from a ruptured acute appendicitis on another, and was discharged with a prescription for Keppra following the onset of seizures during her third admission, with no MRI performed at that time.

During her current admission, the patient was in an altered state and unable to respond to inquiries. Her son, who was present, provided significant background information. He reported that over the past six months, the patient had been experiencing intermittent weakness, which had not been reported during her previous admissions, and had recently begun having episodes resembling seizures. He noted that there was no confusion following these episodes and no urinary or bowel incontinence. The son also mentioned that his mother had recently been prescribed lisinopril for hypertension, and each time she took the medication, she experienced confusion, weakness, fatigue, and seizures.

Upon initial examination, the patient appeared largely obtunded. Her vital signs included a blood pressure of 119/76 mmHg, a pulse of 67 beats per minute, a temperature of 36.9 °C (98.5 °F), a respiratory rate of 25 breaths per minute, and an SpO2 of 100%. The patient exhibited intermittent, asynchronous flailing of her arms. Administration of lorazepam resulted in the termination of these movements.

The neurological assessment was significantly limited due to her unresponsiveness to verbal stimuli; however, she demonstrated movement away from painful stimuli, indicating some level of responsiveness. Reflexes were hyperactive in the bilateral upper extremities, while the lower extremities exhibited good muscle tone and normal reflexes. No nystagmus was observed on eye examination.

The initial diagnostic workup, which included a CBC, complete metabolic panel (CMP), EKG, and chest X-ray, yielded unremarkable results. Urinalysis indicated +1 WBC esterase. A CT scan of the head without contrast revealed chronic ischemic changes but was otherwise unremarkable. The CBC and CMP findings are shown in Table 1 and Table 2, respectively.

**Table 1:** CMP at the time of presentation ALP: alkaline phosphatase; ALT: alanine transaminase; AST: aspartate aminotransferase; CMP: complete metabolic panel; CO₂: bicarbonate; EGFR: estimated glomerular filtration rate; H: high; L: low

Component	Lab value	Reference range
Sodium	145	136-145 mmol/L
Potassium	4.2	3.4-4.9 mmol/L
Chloride	110 (H)	98-107 mmol/L
CO₂	25	21-32 mmol/L
BUN	17	7-25 mg/dL
Creatinine	0.7	0.60-1.30 mg/dL
Glucose	78	74-109 mg/dL
Calcium	9.6	8.6-10.2 mg/dL
AST	10 (L)	13-39 U/L
ALT	6 (L)	7-52 U/L
ALP	45	30-105 U/L
Total protein	8.1	6.4-8.9 g/dL
Albumin	4.5	3.5-5.7 g/dL
Total bilirubin	0.4	0.3-1.0 mg/dL
eGFR	>60.0	>60.0 mL/min/1.73 m²
Anion gap	10	1-11 mmol/L

**Table 2:** CBC at the time of presentation H: high; L: low; MCH: mean corpuscular hemoglobin; MCHC: mean corpuscular hemoglobin concentration; MCV: mean corpuscular volume; MPV: mean platelet volume; RDWSD: red cell distribution width - standard deviation

Component	Lab value	Reference range
RBC	4.57	4.20-5.40 x 10⁶/μL
WBC	5.3	4.5-12.5 x 10³/μL
Hemoglobin	11.9 (L)	12.0-16.0 g/dL
Hematocrit	37.6	36.0-48.0%
MCV	82.3	81.0-99.0 fL
MCH	26.0 (L)	27.0-31.0 pg
MCHC	31.6	31.0-36.0 g/dL
RDWSD	38.6	36.4-46.3 fL
MPV	11.2 (H)	7.4-10.4 fL

Other pertinent labs, including magnesium, ammonia, B12, and a urine drug screen, were all within normal limits.

During the patient’s hospitalization, she received treatment for a UTI with IV ceftriaxone. On the third day of her stay, she began experiencing seizure episodes characterized by jerking movements in her lower extremities. These episodes generally ceased before the administration of antiseizure medication, although some persisted until medication was administered.

The patient exhibited minimal responsiveness to verbal stimuli but responded to sternal rubs during the seizures, without any loss of bladder or bowel control. Following these episodes, she displayed signs of distress, including crying and moaning. A routine EEG was performed, followed by a 24-hour EEG, both of which were negative for epileptiform activity. The patient’s Keppra level was assessed and found to be within the therapeutic range.

In consultation with the neurologist, the Keppra dosage was increased to 750 mg twice daily. As the patient became more alert and oriented, additional inquiries revealed persistent weakness in her lower extremities, a single episode of bladder incontinence at home unrelated to seizures, and intermittent electric-like sensations in her back accompanied by episodes of heat intolerance.

**Figure 1:**
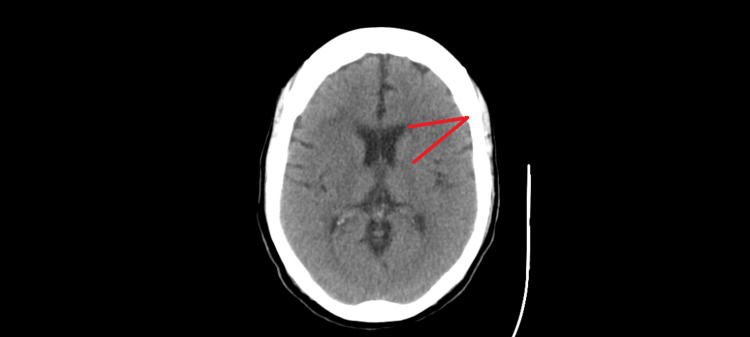
CT head without contrast Two merging lines indicate areas of possible demyelination on an axial view of the brain.

A review of the non-contrast CT scan of the head conducted during this admission, along with her previous scans, suggested a potential pattern of primary or secondary demyelinating processes affecting the corpus callosum and periventricular parenchyma. The CT scan from this admission is shown in Figure 1.

**Figure 2:**
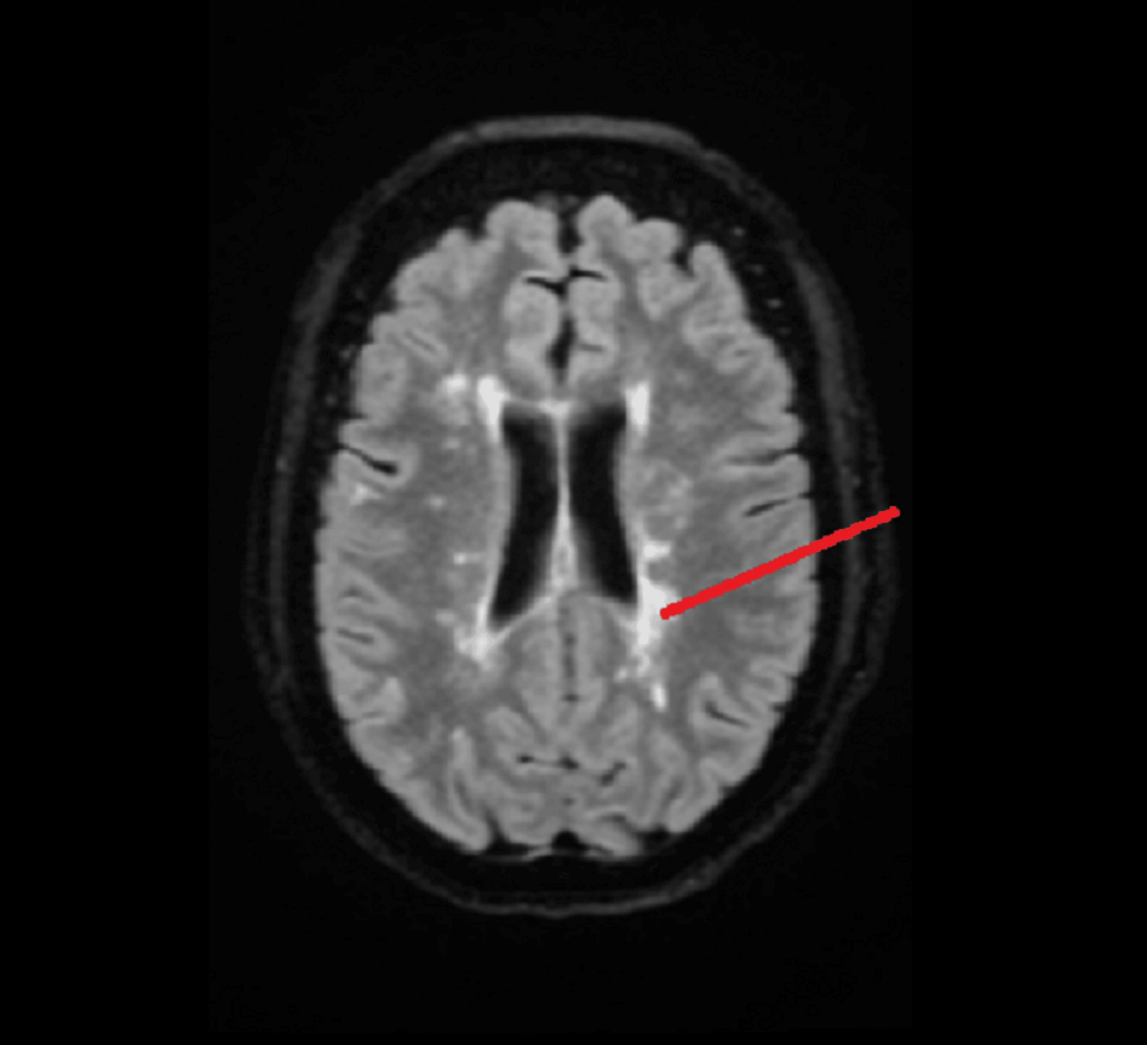
Transverse view of brain MRI The red line points to classic Dawson’s fingers on the brain MRI.

**Figure 3:**
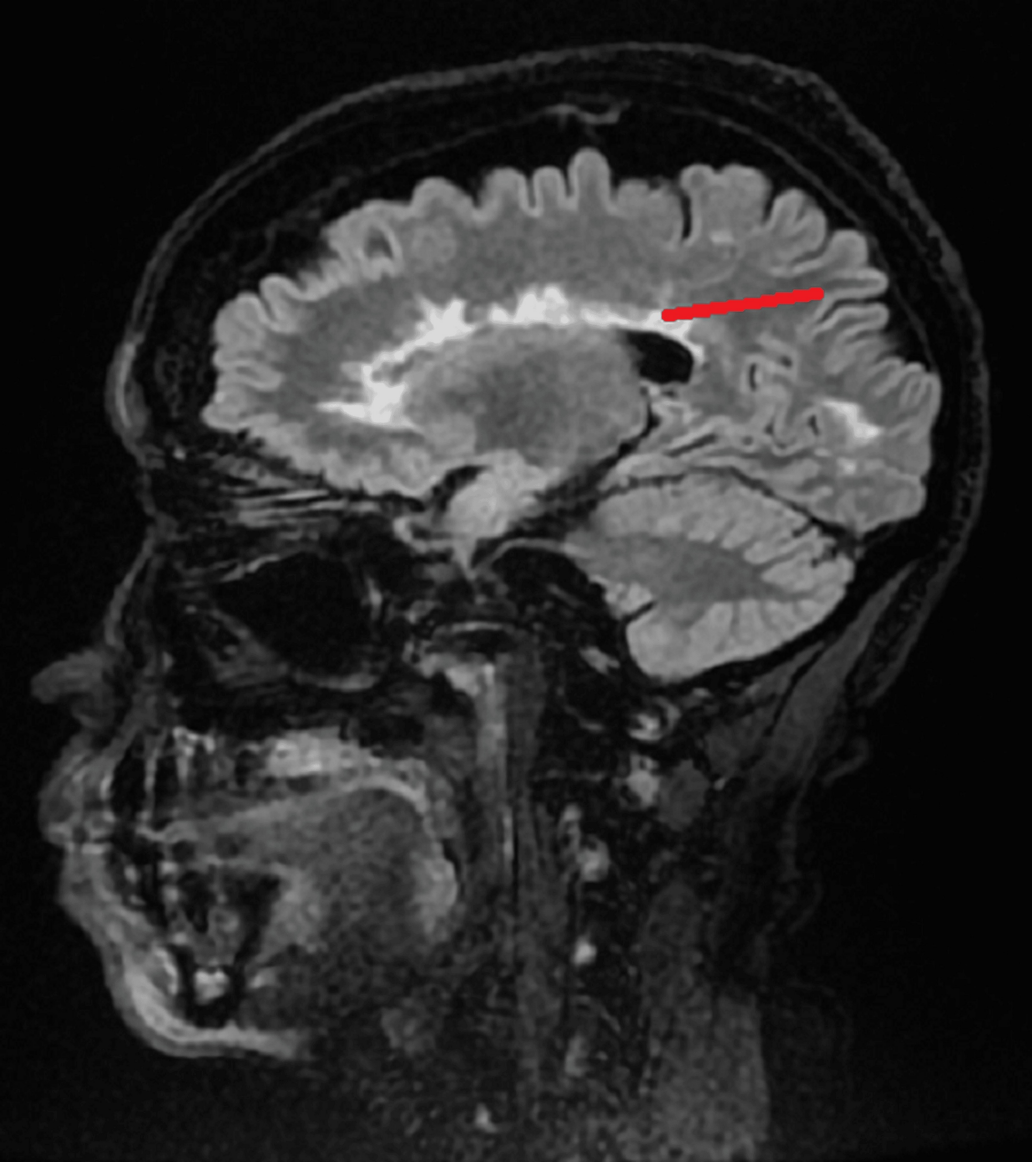
Sagittal view of brain MRI The red line points to Dawson’s finger on the brain MRI.

An MRI of the brain without contrast revealed abnormal findings, including FLAIR hyperintensity in the supratentorial white matter. The pattern was consistent with stigmata of primary demyelinating disease and periventricular white matter changes, including Dawson’s fingers, which are indicative of MS, as shown in Figure 2 and Figure 3.

**Figure 4:**
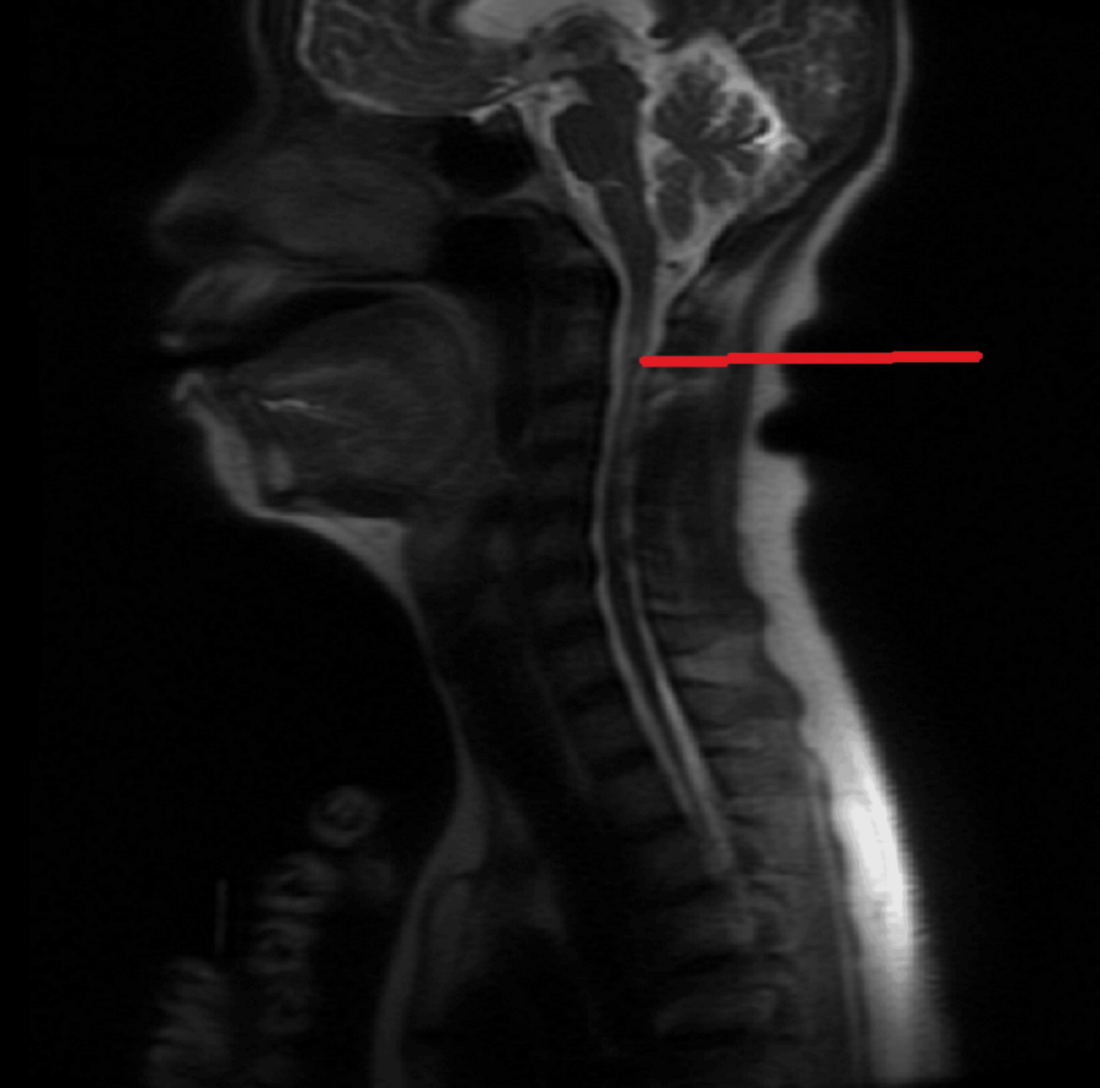
Cervical spine MRI The red line shows lesions within the cervical spinal cord.

An MRI of the spine also revealed a demyelinating lesion in the cervical region, as shown in Figure 4.

Following the diagnosis, the patient was started on IV Solu-Medrol at a dose of 500 mg daily for three days. On the second day of treatment, she experienced another seizure episode characterized by jerking of the lower limbs, followed by drowsiness and confusion. In response, her Keppra dosage was increased to 1,000 mg twice daily. After completing the IV steroid treatment, she was discharged home with noted improvements in lower extremity strength and no further seizures. She returned to baseline with a normal neurological exam, including normal muscle strength and full participation in physical therapy. Before discharge, her antiseizure medication was reduced to 750 mg twice daily. The patient was referred to outpatient neurology for follow-up and ongoing management of her MS. 

## Discussion

MS is an autoimmune disorder primarily affecting the central nervous system, characterized by antibody formation that damages the myelin sheath [3,5]. This damage occurs when perivascular lymphocytes and macrophages infiltrate and degrade the myelin, a protective covering for neurons. Symptoms of MS can vary significantly depending on the location of the lesions and commonly include numbness, tingling, focal weakness, visual disturbances, neurogenic bladder, bowel incontinence, and cognitive deficits [1-3].

The typical onset of MS occurs between the ages of 20 and 40, although cases have been reported in individuals as young as 10 and as old as 50. The condition is more prevalent in women, with a female-to-male ratio of approximately 3:1 [1]. Three main risk factors contribute to the development of MS: immune system characteristics, environmental influences, and genetic predispositions. Studies indicate that individuals living at higher latitudes, further from the equator, have an increased risk of developing MS, possibly due to insufficient vitamin D levels [4,5].

Additionally, certain polymorphisms in immune-related genes, such as HLADR, IL2RA, IL4, IL6, IL12B, IL17R, IRF5, CD24, CD58, and EVI5, show a modest correlation with MS prevalence. Other relevant genetic factors include those regulating vitamin D metabolism (VDR and CYP27B1), mitochondrial DNA variations, genes associated with fibrinolysis (PAI-1), and those involved in CNS functionality and repair mechanisms (ApoE and DPP63) [4-7].

MS is classified into four main types. The most common type is relapsing-remitting (RR) MS, affecting approximately 70-80% of patients. This type is characterized by episodes of new or returning neurological symptoms typically lasting from 24 to 48 hours, although the onset can extend over several days to weeks [3,5]. Primary progressive MS, occurring in about 15-20% of patients, is marked by a continuous decline in neurological function from symptom onset without relapses.

Secondary progressive MS follows an initial RR phase and involves gradual neurological deterioration, with some individuals experiencing additional relapses [1,2,5]. Progressive-relapsing MS, the least common type, represents approximately 5% of cases and involves a steady decline accompanied by relapses.

Our patient’s MS symptoms began at the age of 55, with a formal diagnosis made at 56, primarily due to the atypical presentation of her symptoms and the later age of onset. While seizures occur in approximately 3-4% of individuals with MS, they are generally not considered a primary symptom. Diagnosing MS follows the McDonald criteria, which require evidence of CNS damage that is both spatially and temporally disseminated, meaning neurological deficits must be present in at least two separate areas of the CNS or occur at different intervals [5].

MRI evaluations of our patient, shown in Figure 2, Figure 3, and Figure 4, revealed lesions in the periventricular area with the characteristic Dawson’s fingers pattern, along with lesions in the cervical spinal cord.

Following diagnosis, the patient underwent treatment with a high-dose steroid regimen of 500 mg of IV methylprednisolone administered daily for three days, consistent with the recommended dosage of 500-1,000 mg per day for three to five days. By the second day of treatment, significant improvements were noted in fatigue, bilateral lower extremity weakness, and spasms. Additionally, increasing the Keppra dosage successfully eliminated the daily seizures.

Identifying the underlying cause of seizures is essential for effective treatment. In this case, despite the patient receiving a therapeutic dosage of Keppra, seizures remained uncontrolled until the diagnosis and proper management of the underlying condition, MS, were established.

## Conclusions

Seizures, although possible in individuals with MS, are considered an uncommon and atypical manifestation of the condition. Thorough examination and clinical evaluation are essential for the accurate diagnosis and management of MS, ensuring timely treatment. Effective management following acute flare-ups is often critical to prevent recurrent episodes. The presence of seizures in patients with MS adds complexity to the disease’s presentation. While seizures may develop years after an initial MS diagnosis, in this case, seizure symptoms emerged alongside other MS symptoms, complicating the differentiation between the two conditions and subsequently hindering treatment. Although seizures in individuals with MS are rare, MS should still be considered as a potential underlying cause when seizures occur, and a follow-up MRI is recommended to support prompt diagnosis. The McDonald criteria remain a valuable tool for diagnosing MS. In most cases, lumbar puncture and cerebrospinal fluid analysis for oligoclonal bands are unnecessary when MRI findings are available and the McDonald criteria can be applied effectively.

Managing seizures associated with MS can be successfully achieved with AEDs alongside appropriate treatment for MS. In this case, administering a pulse dose of steroids (500 mg of Solu-Medrol daily for three days) to address the acute MS flare significantly contributed to controlling the seizure episodes. Following treatment for the acute flare, the Keppra dosage was reduced, and the patient was discharged with a return to her baseline neurological function.
